# Lower Cortisol and Dehydroepiandrosterone Sulfate and Higher Food Addiction in Childhood Obesity: Associations With Stress and Dietary Parameters

**DOI:** 10.1210/jendso/bvaf011

**Published:** 2025-02-10

**Authors:** Rúbia Cartaxo Squizato de Moraes, Thallyta Alanna Ferreira Viana, Joicy Karla Grangeiro Pereira, Paulo César Trindade da Costa, Davyson Barbosa Duarte, Lydiane de Lima Tavares Toscano, Manuel Francisco de Araújo Lima, Melyssa Kellyane Cavalcanti Galdino, Joelma Rodrigues de Souza, Francisco Antônio de Oliveira Júnior, Adélia da Costa Pereira de Arruda Neta, José Luiz de Brito Alves, Vinícius José Baccin Martins

**Affiliations:** Department of Nutrition, Federal University of Paraíba, João Pessoa, Paraíba 58051-900, Brazil; Department of Nutrition, Federal University of Paraíba, João Pessoa, Paraíba 58051-900, Brazil; Department of Nutrition, Federal University of Paraíba, João Pessoa, Paraíba 58051-900, Brazil; Department of Nutrition, Federal University of Paraíba, João Pessoa, Paraíba 58051-900, Brazil; Laboratory of Nutrition, Physical Activity and Phenotypic Plasticity, Academic Center of Vitória, Federal University of Pernambuco, Vitória de Santo Antão, Pernambuco 55608-680, Brazil; Department of Physical Education, Federal University of Paraíba, João Pessoa, Paraíba 58051-900, Brazil; Department of Psychology, Federal University of Paraíba, João Pessoa, Paraíba 58051-900, Brazil; Department of Psychology, Federal University of Paraíba, João Pessoa, Paraíba 58051-900, Brazil; Department of Physiology and Pathology, Federal University of Paraíba, João Pessoa, Paraíba 58051-900, Brazil; Department of Physiology and Pathology, Federal University of Paraíba, João Pessoa, Paraíba 58051-900, Brazil; Center for Food Studies and Research, State University of Campinas, Campinas, São Paulo 13083-852, Brazil; Department of Nutrition, Federal University of Paraíba, João Pessoa, Paraíba 58051-900, Brazil; Department of Physiology and Pathology, Federal University of Paraíba, João Pessoa, Paraíba 58051-900, Brazil

**Keywords:** childhood obesity, cortisol, food addiction, DHEA, NOVA classification, stress

## Abstract

**Context:**

Obesity has been associated with changes in cortisol and dehydroepiandrosterone (DHEA) sulfate concentrations and increased stress levels and food addiction.

**Objectives:**

We explored changes in morning salivary cortisol and DHEA in childhood obesity and their associations with body composition, metabolic profile, food addiction, food consumption, and stress in a cross-sectional study.

**Methods:**

Children aged 7 to 12 years of both sexes were allocated into 2 groups according to body mass index-for-age: control group (n = 60) or obesity group (n = 98). Anthropometric, body composition, serum glucose, insulin, lipid profile, and DHEA were measured. Saliva was collected at different times to measure morning salivary cortisol concentrations. Food addiction, food consumption, and stress were assessed using questionnaires.

**Results:**

Lower DHEA [1.04 (0.87-1.25) ng/mL vs 1.65 (1.30-2.07) ng/mL, *P* = .002] and salivary cortisol (6:00 Am: 1.17 ± 0.89 vs 1.45 ± 0.82 nmol/L, 6:30 Am: 1.53 ± 0.68 vs 1.83 ± 0.70 nmol/L, 7:30 Am: 0.72 ± 0.99 vs 1.31 ± 0.94 nmol/L, *P*-value of time < 0.001 and *P*-value of group = .002) were observed in children with obesity compared to the control. DHEA correlated negatively with waist circumference (r = −0.20, *P* < .05), body mass index-for-age(BMI-Z) (r = −0.21, *P* < .01), and weight (r = −0.25, *P* < .01). DHEA showed a positive correlation with the cortisol area under the curve (r = 0.29, *P* = .002). Food addiction was positively correlated with waist circumference (r = 0.21, *P* < .01), BMI-Z (r = 0.22, *P* < .01), body weight (r = 0.20, *P* < .05), total energy intake (r = 0.20, *P* < .05), and lipids (r = 0.24, *P* < .01).

**Conclusion:**

Children with obesity showed lower concentrations of salivary cortisol and DHEA and higher food addiction compared to control children. These changes may contribute to the development of chronic diseases over time.

The prevalence of obesity has tripled in recent decades, partly due to economic development and lifestyle changes [1]. Globally, more than 390 million children and adolescents aged 5 to 19 years were overweight in 2022, including 160 million with obesity [2]. In Brazil, about 10% of children and adolescents are affected by obesity [3]. The consequences of obesity in infancy and early childhood impact nearly every aspect of health and can lead to chronic diseases in adulthood, resulting in significant impairment and disability [4].

Consumption of highly palatable foods has been shown to stimulate stress hormones that alter limbic (emotional) and striatal (motivational) pathways, promoting behavioral signs of addiction and excessive food intake [5]. The primary physiological pathway involves cortisol, which stimulates fat storage, insulin resistance [6] and changes in dietary behavior through increased reward sensitivity (dopamine and opioid systems) and increased appetite (arcuate nucleus in the hypothalamus) [7]. These physiological pathways are associated with food addiction [8], which involves changes in eating behaviors driven by the supraphysiological consumption of high-energy and palatable foods, characterized by dependence, impulsivity, and compulsion [9].

Different clinical observational studies have shown both positive and negative associations between body mass index (BMI) and cortisol concentrations. Some studies demonstrate a positive relationship between hair cortisol and obesity [10, 11], while others indicate a decrease in salivary or plasma cortisol concentrations [12, 13].

In addition to cortisol, the adrenal gland secretes the majority of dehydroepiandrosterone (DHEA) [14] and its sulfated form, dehydroepiandrosterone sulfate (DHEAS), which are the most abundant steroid hormones in humans. Although physiological effects have been described for both [15], DHEAS is typically metabolized to DHEA within cells before exerting its physiological effects [16, 17]. DHEA has been reported to have beneficial effects in animal models of diabetes mellitus and obesity, including reducing gluconeogenesis in the liver [18]. In some countries, DHEA is classified as a dietary supplement, encouraging its consumption. Low concentrations of DHEA have been linked to greater coronary stenosis and insulin resistance, while DHEA supplementation has been associated with a reduction in body fat percentage [19]. Conversely, in children with obesity, greater central adiposity and rapid weight gain are associated with increased DHEAS concentrations in 7-year-old children [20].

Although individuals with obesity have a higher prevalence of food addiction, findings on cortisol concentrations in children with obesity are contradictory. Stress in these children may increase cortisol concentration. In addition, few studies have analyzed DHEA and cortisol concentrations in this population. Both cortisol and DHEA have pleiotropic effects with physiological impacts on various systems, and changes in these hormones are linked to the development of long-term diseases. Therefore, our primary aim was to analyze morning salivary cortisol and DHEA concentrations in children with obesity and compare them with those of children with normal weight. Our secondary aim was to examine the associations of salivary cortisol and DHEA with body composition, metabolic profile, food addiction, food consumption, and stress.

## Methods

### Study Design and Population

A cross-sectional study was conducted with children and adolescents aged 7 to 12 years, of both sexes, from 7 public schools located in a low-income area of João Pessoa City, Brazil. The children included in the study were allocated as follows: (1) control group, body mass index-for-age (BMI-Z) >−1.0 and <1.0 Z-score and (2) obesity group, BMI-Z >2.0 Z-score. A height-for-age Z-score greater than −2.0 was used to indicate adequate height in both groups. Children with obesity were matched with control children based on age and sex; for each child with obesity, a normal-weight child of the same age and sex was invited to participate.

Data on BMI-Z and height-for-age on 2222 students (aged 5-17) of the 7 public schools of João Pessoa were analyzed. Of these, 746 did not meet the age criteria, 29 (1%) students were identified as stunted (height-for-age < −2.0 Z-score), 54 (3.7%) were underweight (BMI-Z < −2.0 Z-score), and 250 (17.3%) were overweight (BMI-Z > 1.0 and <2.0 Z-score); these children and adolescents were not invited to participate in the study. From 248 (17.2%) children with obesity, 8 were also not invited due to psychological or behavioral disorders. All students who met the criteria for the obesity group (n = 240) were invited to participate, but 142 did not agree to participate. The obesity group comprised 98 children (51 girls and 47 boys), while the control group consisted of 60 children (39 girls and 21 boys). None of the participants in this study were using any medication or had autoimmune, cardiovascular, renal, or endocrine diseases (exclusion criteria) that could interfere with the analysis.

The present study was approved by the Research Ethics Committee of the Federal University of Paraíba (CAAE 53905321.9.0000.5188), and all procedures were conducted following the Declaration of Helsinki. Consent forms were signed by all parents and participants before they participated in the research.

### Protocol

The research protocol was conducted in 3 stages. In the first stage, anthropometric data (height-for-age and BMI-Z scores) from 7 public schools were analyzed. Based on these measurements, children who met the anthropometric criteria were selected and invited to participate in the study. After obtaining consent from parents or guardians, a health history was obtained from the guardians to ensure that their child met the inclusion/exclusion criteria for the study. The second stage involved blood collection, anthropometric measurements, body composition assessments, and food consumption surveys. The third stage included saliva sample collection, assessment of child stress levels, a second food consumption survey, and evaluation of food addiction. These stages were conducted on separate, nonconsecutive days. For the second and third stages, children and adolescents were instructed to fast overnight for 12 hours. After completing the anthropometric evaluations and blood samples or saliva samples (depending on the stage), all participants were provided with breakfast.

### Anthropometric Assessment and Body Composition

Students were weighed using a digital scale platform (Omron—HBF—514) with a capacity of 150 kg and a precision of 100 g. Height was measured using a stadiometer (Alturaexata) with a precision of 1 mm. BMI-Z and height-for-age were calculated using WHO AnthroPlus software (v1.0.4 WHO). Waist circumference (WC) and hip circumference were evaluated in the standing position, with the abdomen relaxed and the arms at the sides of the body, using an inelastic tape measure with an accuracy of 1 mm. Waist-to-hip ratio was determined by the dividing waist circumference by hip circumference in centimeters. All anthropometric measurements were performed in triplicate, and the arithmetic mean was recorded.

Triceps and subscapular skinfolds were measured using an adipometer (Sanny—AD1009), and the body fat percentage (BFP) was calculated according to Slaughter [21]. Measurements were made in triplicate, and the arithmetic mean was recorded.

### Biochemical and Hormonal Analysis

Blood samples were obtained after an overnight fast of 12 hours and centrifuged at 3000 rpm for 15 minutes. Serum and plasma were stored at −80 °C for further analysis. Total cholesterol, high-density lipoprotein cholesterol (HDL), low-density lipoprotein cholesterol (LDL), triglycerides, and glucose concentrations were analyzed using the enzymatic colorimetric method on an automated analyzer (Chem Well T—Labtest), following the manufacturer's recommendations (Labtest, Mins Gerais, Brazil). Insulin (Elabscience, Houston, TX, USA, RRID:AB_3668893), and DHEA concentrations (Finetest, Wuhan, China, RRID:AB_3668897) were analyzed by ELISA, according to the protocol recommended by the manufacturer, with a sensitivity of 0.47 μIU/mL and 0.938 mg/mL and intra-assay and interassay coefficients of variation below 10% and 6%, respectively.

Insulin resistance was assessed using the homeostasis model assessment of insulin resistance (HOMA-IR) calculator provided by Oxford [22].

### Salivary Cortisol

Three saliva samples were collected from the children using the salivette® (Sarstedt, Leicester, UK) at 3 different times: time 1, immediately upon waking up (6:00 Am); time 2, 30 minutes after waking up (6:30 Am); and time 3, 90 minutes after waking up, upon arriving at school in the morning (7:30 Am). Before the collection, both the child and their guardian were instructed to fast from food or drink, to refrain from exercise the day before, and to avoid brushing their teeth. Before each collection, the mouth was rinsed with water to remove any residues present on the oral mucosa. Collection was contraindicated in the presence of cuts, wounds, or blood in the mouth. Samples were then centrifuged (1500 g, 4 °C, 20 minutes) and stored at −20 °C until analysis using an ELISA kit following the manufacturer's instructions (Arbor Assays, Ann Arbor, MI, USA, RRID:AB_2893032) with a sensitivity of 27.6 pg/mL and intra-assay and interassay coefficients of variation below 15%. The area under the curve (AUC) of cortisol was calculated in the Prisma software (version 8.0) using the 3 times of salivary cortisol in nanomoles per liter. Samples whose intervals between time were not punctual or children who did not have all 3 samples collected were excluded from this analysis.

### Food Addiction

The Yale Food Addiction Scale for Children, developed by Gearhardt [23], aims to investigate food addiction in children using a questionnaire comprising 25 items grouped into 7 symptoms criteria for diagnosing substance dependence based on the *Diagnostic and Statistical Manual of Mental Disorders*. These criteria include tolerance; withdrawal; consuming the substance in larger amounts or over a longer period than was intended; persistent desire or unsuccessful effort to cut down or control substance use; spending a great deal of time in activities necessary to obtain or use the substance or to recover from its effects; giving up social, occupational, or recreational activities because of substance use; and continuing the substance use with the knowledge that it is causing or exacerbating a persistent or recurrent physical or psychological problem. To be classified as food addiction, clinical distress and 3 or more symptoms are necessary. The Yale Food Addiction Scale for Children, validated in Brazilian children and adolescents [24], with a Cronbach's alpha of .83, was used in this study to identify the presence of food addiction among the participants.

### Food Consumption

Information on the food intake of the children was collected through the 24-hour food recall on 2 different days: 1 response for the weekend and the other for a weekday [25], to estimate intrapersonal dietary variability and enhance accuracy [26]. At least 1 24-hour food record was self-reported with the help of mothers or other caregivers. During interviews conducted by trained nutritionists, a Global Diet photo album was used to define portion sizes. The foods and beverages consumed during the previous 24 hours were reported, including details such as preparation methods, brand names of processed foods, and portion sizes. Data from the food record were tabulated using the Brasil Nutri® software, chosen for its compatibility with Brazilian food composition tables used in the Food Research Family Budgets [26]. Usual energy and nutrient intakes were estimated using the Multiple Source Method, a statistical method for estimating habitual dietary intake based on 2 or more short-term measurements, such as 24-hour dietary recalls [27]. Reported foods were classified by degree of processing using the NOVA classification, which categorizes foods as fresh and minimally processed (UMPF), culinary ingredients, processed, and ultra-processed [28]; however, in the present study, culinary ingredients were not included. UMPF were defined as foods consumed in their natural state or subjected to minimal processing methods that largely preserve the food matrix, such as removing parts, drying, toasting, boiling, pasteurization, refrigeration, freezing, vacuum packaging, or nonalcoholic fermentation, such as fruit, vegetables, grains, seeds, fresh meat, fish, viscera, eggs, milk, roots, and tubers. Processed foods were foods with added substances to extend shelf life or prevent microbial proliferation using methods like canning and bottling, nonalcoholic fermentation, boiling, or baking. Examples included bread, cheese, yogurt, cake, cookies, canned or bottled vegetables and legumes in brine, salted or sugared nuts and seeds, fruits in syrup, ultra-processed foods, and foods that undergo multiple stages of industrial processing, often involving the use of additives like flavorings, colorings, sweeteners, and emulsifiers to enhance palatability. Examples include soft drinks, reconstituted fruit juices, dairy drinks, energy drinks, flavored yogurt, filled cookies, snacks, instant noodles, candies, poultry and fish nuggets and sticks, sausages, reconstituted meat products, cured meats, and preprepared dishes like pies, pasta, and pizzas with additives.

### Stress

Stress was evaluated using the Lipp Stress Symptom Inventory, validated for Brazilian Portuguese by Lipp and Guevara [29]. This inventory has 35 items grouped into 4 factors: physical reaction, psychological reaction, psychological reaction with a depressive component, and psychophysiological reactions. Trained psychologists administered the inventory to diagnose stress in children.

### Statistical Analysis

The variables were assessed for normality using the Shapiro–Wilk test. Variables that did not follow a normal distribution were log-transformed when applicable. Anthropometric, biochemical, hormone, and food consumption data were analyzed using *t*-tests. ANCOVA, adjusting for sex, was employed for body composition data. A mixed between-within subjects ANOVA was used to evaluate morning salivary cortisol concentrations between times, groups, and interactions between time and group. The chi-square test was used to analyze stress and food addiction. Anthropometric, hormone, stress, and food addiction data were correlated with age, body composition, biochemical, and food consumption using either Pearson's or Spearman's correlation analysis, as recommended, with Benjamini–Hochberg correction for multiple tests. All data were expressed as mean and SD, geometric mean and confidence interval, or absolute values and percentages. Statistical analysis was conducted using SPSS software (IBM version 20.0).

A posteriori power calculation (GPower 3.1.9.7, University of Kiel, Germany) was performed using DHEA as the primary outcome, considering a sample size of 158 subjects (60 in the control group and 98 in the group with obesity), with an effect size of 0.51 and an α of .05, which would provide a power of over 85%.

## Results

Table 1 shows the anthropometric characteristics and body composition of the 2 groups. No differences in sex and age were found between the groups. Weight, height, BMI-Z, height-for-age, WC, hip circumference, waist-to-hip ratio, BFP, and absolute body fat were significantly higher in the obesity group compared to the control group.

**Table 1.** Anthropometric characteristics and body composition of the participants

	Groups				
	Control (n = 60)		Obesity (n = 98)		*P*-value
	n or mean	% or SD or CI	n or mean	% or SD or CI	
Girls	39	65	51	52	.110
Boys	21	35	47	48	
Age (years)	9.58	1.25	9.27	1.18	.114
Weight (kg)	30.87	6.61	50.60	11.34	<.001
Height (cm)	137.50	9.70	141.70	8.64	.005
BMI-Z (Z-score)	−0.23	0.78	2.83	0.74	<.001
Height-for-age (Z-score)	0.23	0.85	1.25	1.02	<.001
WC (cm)(	58.79	5.19	79.59	9.34	<.001
HC (cm)	71.70	6.66	90.59	8.60	<.001
WHR (cm)	0.82	0.04	0.87	0.06	<.001
Body fat (%)	21.49	19.70-23.29	36.03	34.65-37.41	<.001
Body fat (kg)	6.88	5.20-8.56	18.69	17.40-19.98	<.001

Chi-square test was applied for sex analysis. Data are shown as n and percent.

*t*-test was applied for age and anthropometric parameters. Data are shown as mean and SD.

Analysis of covariance adjusted for sex was applied for body composition parameters. Data are presented as mean and CI.

Abbreviations: BMI-Z, body mass index-for-age; CI, confidence interval; HC, hip circumference; WC, waist circumference; WHR, waist-hip ratio.

Table 2 shows the metabolic and hormonal profiles of the children. Children with obesity had lower HDL and DHEA and higher triglycerides, glucose, insulin concentration, and HOMA-IR than the control group. No significant differences were found in total cholesterol and LDL between groups.

**Table 2.** Metabolic and hormonal profile of the groups

	Groups				
	Control (n = 60)		Obesity (n = 98)		*P*-value
	Mean	SD or CI	Mean	SD or CI	
Total cholesterol (mmol/L)	4.62	0.78	4.48	0.79	.258
HDL (mmol/L)	1.51	0.34	1.29	0.35	<.001
LDL (mmol/L)	2.40	0.58	2.41	0.48	.955
Triglycerides (mmol/L)	0.81	0.36	1.06	0.46	.001
Glucose (mmol/L)^*a*^	4.34	4.22-4.44	4.48	4.39-4.62	.032
Insulin (pmol/L)^*a*^	80.60	68.0-96.5	149.83	130.26-172.34	<.001
HOMA-IR^*a*^	1.43	1.21-1.70	2.61	2.29-2.97	<.001
Dehydroepiandrosterone (ng/mL)^*a*^	1.65	1.30-2.07	1.04	0.87-1.25	.002

T-test was applied for metabolic and hormonal parameters. Data are presented as mean and SD or CI.

Abbreviations: CI, confidence interval; HDL, high-density lipoprotein cholesterol; HOMA-IR, homeostasis model assessment of insulin resistance; LDL, low-density lipoprotein cholesterol.

^*a*^Logarithmically transformed. Data are presented as geometric mean and CI.

**Figure 1.**
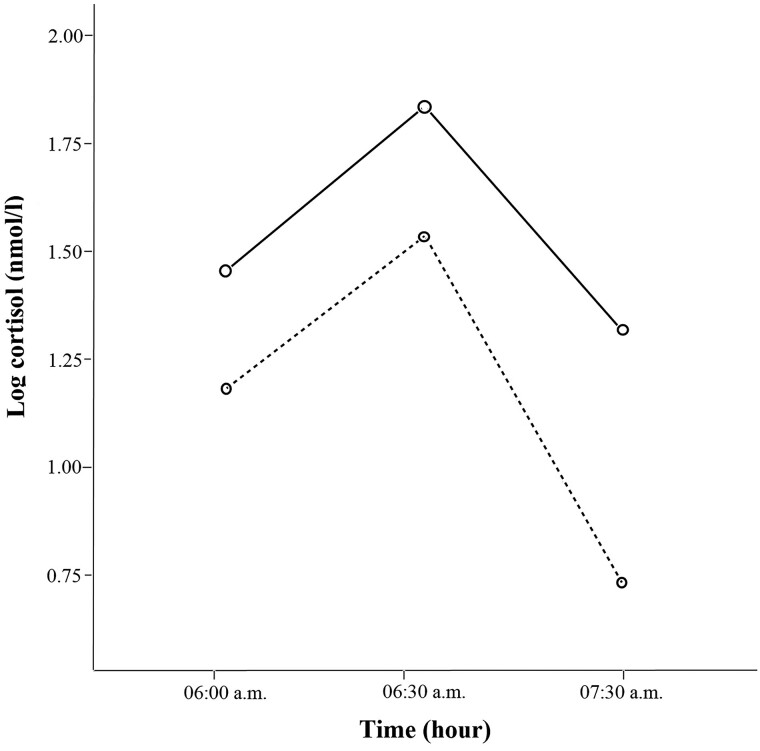
Morning salivary cortisol measurements were taken at 3 time points: 6:00 Am (immediately upon waking), 6:30 Am (30 minutes after waking), and 7:30 Am (90 minutes after waking). Cortisol concentrations significantly decreased over time in both groups. However, children with obesity exhibited lower cortisol concentrations compared to the control group. No significant interaction between time and group was observed. Mixed between-within subjects ANOVA. Control group 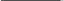 (continuous line); obesity group 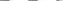 (dashed line). Wilk's lambda for time: F = 26.83, *P* < .001; Wilk's lambda for interaction between time and group: F = 1.16, *P* = .316. *P*-value of group = .002.

Differences in morning salivary cortisol measurements between groups and times are shown in Fig. 1. Salivary cortisol concentrations differed significantly across the 3 measurement times (*P* < .001), with children with obesity having lower salivary cortisol concentrations compared to the control group (*P* = .002). No significant interaction between time and group was found (*P* = .316).

**Figure 2.**
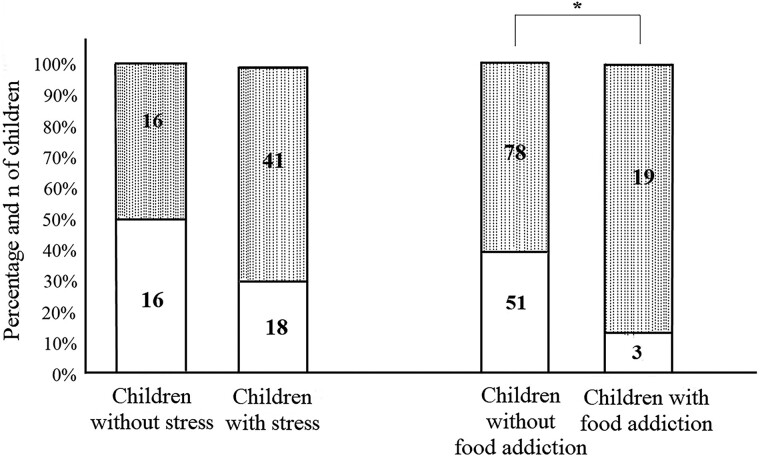
Stress and food addiction between groups. No differences were found in the proportion of children with stress between the groups; however, the children with obesity had a higher prevalence of food addiction. Chi-square test. Data are shown as n. Control group 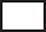 (white box), obesity group 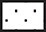 (dotted box). *P*-value of stress = .066. *P*-value of food addiction = .028.

The proportion of children with stress in the obesity group was not different from the control group (*P* = .066, Fig. 2). However, children with obesity showed a high proportion of food addiction (*P* = .028, Fig. 2).

No significant differences were found in the density of the food groups according to the NOVA classification and in total energy consumption and macronutrients (Table 3).

**Table 3.** Food consumption using the NOVA classification system and macronutrients

	Group				*P*-value
	Control (n = 60)		Obesity (n = 98)		
	Mean	SD	Mean	SD	
Unprocessed or minimally processed foods (%)	41.77	10.46	43.95	10.80	.230
Processed foods (%)	20.50	9.76	21.64	8.92	.517
Ultra-processed foods (%)	44.25	11.22	41.43	10.88	.133
Total Energy (Kcal)	1940.82	490.19	1930.33	429.03	.891
Protein (g)	72.60	23.25	73.48	18.26	.797
Carbohydrate (g)	262.35	69.96	261.76	69.18	.960
Lipids (g)	71.28	14.89	70.23	12.51	.643

T-test was applied for food consumption parameters. Data are shown as mean and SD.

Table 4 shows the correlation of anthropometric, body composition, hormonal, metabolic, stress, food addiction, and food consumption variables. BMI-Z, WC, and BFP showed a negative correlation with HDL and a positive correlation with triglycerides, insulin, and HOMA-IR. BFP showed a positive correlation with LDL, and WC positively correlated with glucose. DHEA showed a negative correlation with WC, BMI-Z, weight, and glucose and a positive correlation with total cholesterol, HDL, and UMPF. The cortisol AUC was negatively correlated with WC, BMI-Z, and weight. Stress correlated negatively with HDL, and food addiction showed positive associations with WC, BMI-Z, weight, total energy intake, and lipids. No correlations were found between stress and food addiction with food consumption according to the NOVA classification.

**Table 4.** Correlation of anthropometric, body composition, hormonal, metabolic, stress, food addiction, and food consumption variables

	BMI-Z (score z)	WC (cm)	BFP	DHEA (ng/mL)	Cortisol AUC	Stress	Food addiction
Age (years)	−0.14	0.17	0.03	−0.08	0.00	−0.10	0.00
WC (cm)	0.88*^a^*	1	0.80*^a^*	−0.20*^b^*	−0.24*^b^*	0.15	0.21*^b^*
BMI-Z (Z-score)	1	0.88*^a^*	0.82*^a^*	−0.21*^b^*	−0.29*^b^*	0.18	0.22*^b^*
Weight (kg)	0.80*^a^*	0.91*^a^*	0.78*^a^*	−0.25*^a^*	−0.27*^b^*	0.15	0.20*^b^*
Total cholesterol (mmol/L)	0.00	−0.01	0.04	0.27*^a^*	0.08	−0.03	−0.15
HDL (mmol/L)	−0.29*^a^*	−0.29*^a^*	−0.27*^a^*	0.34*^a^*	0.17	−0.27*^b^*	−0.18
LDL (mmol/L)	0.09	0.07	0.16	0.07	0.06	0.07	−0.05
Triglycerides (mmol/L)	0.29*^a^*	0.31*^a^*	0.31*^a^*	−0.05	−0.15	0.10	0.07
Glucose (mmol/L)	0.14	0.19	0.12	−0.20*^b^*	−0.09	0.00	−0.03
Insulin (pmol/L)	0.52*^a^*	0.56*^a^*	0.52*^a^*	−0.02	−0.24	0.19	0.11
HOMA-IR	0.52*^a^*	0.56*^a^*	0.52*^a^*	−0.03	−0.24	0.18	0.11
UMPF (%)	0.06	0.08	0.04	0.22*^b^*	−0.04	−0.03	−0.11
PF (%)	0.04	0.05	0.05	−0.13	−0.05	−0.05	−0.10
UPF (%)	−0.05	−0.04	0.00	−0.14	0.09	−0.01	0.06
Total Energy (Kcal)	0.08	0.04	0.01	−0.13	−0.03	0.12	0.20*^b^*
Protein (g)	0.00	−0.05	−0.07	−0.05	0.06	0.01	0.15
Carbohydrate (g)	0.00	−0.03	−0.06	−0.11	0.02	0.14	0.19
Lipids (g)	−0.01	−0.05	−0.04	−0.08	0.01	0.09	0.24*^b^*

Pearson correlation was applied for BMI-Z, WC, BFP, DHEA, and cortisol AUC.

Spearman correlation was applied for stress and food addiction.

*P*-values were adjusted for multiple testing using the Benjamini–Hochberg correction.

Abbreviations: AUC, area under the curve; BFP, body fat percentage; BMI-Z, body mass index-for-age; DHEA, dehydroepiandrosterone; HDL, high-density lipoprotein cholesterol; HOMA-IR, homeostasis model assessment of insulin resistance; LDL, low-density lipoprotein cholesterol; PF, processed foods; UMPF, unprocessed and minimally processed foods; UPF, ultra-processed foods; WC, waist circumference.

^*a*^ *P* < .01.

^*b*^ *P* < .05.

DHEA also showed positive correlations with salivary cortisol at 30 minutes after waking up (0.27, *P* = .002), salivary cortisol at 90 minutes after waking up (0.27, *P* = .003), and the AUC of the cortisol (0.29, *P* = .002), however, DHEA did not show a correlation with stress (−0.13, *P* = .197) (data not show in the table).

## Discussion

The findings of this study show adverse changes in lipid and carbohydrate metabolism in children with obesity, including higher concentrations of triglycerides, glucose, insulin, and HOMA-IR and lower concentrations of HDL. These changes in biochemical parameters and hormonal profiles in children with obesity, compared to control children, may indicate an association with the development of long-term diseases [30]. Additionally, children with obesity had lower concentrations of DHEA and morning salivary cortisol, along with a higher proportion of food addiction. This food addiction was positively associated with BMI-Z, body weight, and consumption of total calories and lipids in our study.

In prepubertal children with obesity, higher concentrations of DHEAS have been observed even after weight loss, while cortisol and testosterone concentrations, initially higher than in normal weight controls, decreased after weight loss [31]. It has been suggested that obesity promotes permanent changes in the adrenal gland, as DHEAS concentrations do not decrease after weight loss. At baseline, the severity of obesity in these studies was similar to that in the present study (BMI-Z 2.33 vs 2.83, respectively). Children with obesity of both sexes at Tanner I had twice the risk of high DHEAS at age 7, with positive associations between DHEAS and BMI, as well as WC [32]. Another cohort of children 11 years old, with anthropometric results similar to our study (BMI-Z 2.12 vs 2.83, respectively, and WC 92.83 vs 79.59), showed positive correlations between DHEAS concentrations and WC [33]. In the present study, lower DHEA concentrations and negative associations between DHEA and WC, BMI-Z, and weight were found. Although attenuation of DHEA and DHEAS concentrations has been described in response to psychosocial stress in young adults with depressive symptoms but normal weight [34], children with obesity in our study did not show a higher prevalence of stress, and no correlation was found between stress level and WC, BMI-Z, or weight. In the present study, the prevalence of stress did not differ between groups, which may be attributed to the age of the children. It is known that emotional changes and higher levels of stress typically occur during the pubertal period [35]. These findings suggest lower DHEA availability in children with obesity compared with control children, and this decrease is in line with the physiological effects of DHEA.

DHEA has pleiotropic effects, including improved endothelial function and cellular immunity, reduced inflammation, and amelioration of atherosclerosis [36]. It is also used as an antiobesity supplement [37]. The positive correlation between DHEA and HDL in this study can contribute to the improvement of atherosclerosis. Experimental studies with rats consuming foods containing DHEA showed reduced body weight gain and lower steatosis scores compared to diets without DHEA [37]. Additionally, DHEA showed a negative correlation with glucose in the present study, which can be partially explained by its effect on the liver and muscles, increasing insulin sensitivity [18]. In muscle cell culture, DHEA stimulates the expression of GLUT 4 [38]. Furthermore, these findings help explain, at least in part, the negative correlations between DHEA and anthropometric parameters, as DHEA has been associated with protection against obesity [17, 18]

Although the regulatory mechanisms of DHEA remain unclear [39], DHEA secretion is stimulated by cortisol at physiological concentrations [40]. In culture, human cells treated with increasing doses of cortisol showed a dose-dependent increase in DHEA secretion from 50 μM, with this secretion linked to the competitive inhibition of 3βHSD2 activity. These findings are in line with the positive correlation between DHEA and cortisol concentrations observed 30 and 90 minutes after waking, as well as the AUC of cortisol found in the present study. Additionally, both cortisol and DHEA concentrations were lower in children with obesity. However, fewer studies have examined the association between morning cortisol patterns and obesity in childhood, and the results are divergent.

One study focused on adolescents found that blunted salivary cortisol was associated with increased BMI measures both concurrently and longitudinally [41]. Another study comparing pre- and middle-school-aged children found that overweight and obesity at preschool age predicted lower future morning concentrations of salivary cortisol and blunted cortisol reactivity [42]. These authors suggested a downregulation of the hypothalamic-pituitary-adrenal axis with excess adipose tissue [41, 42]. In the present study, children with obesity exhibited lower cortisol concentrations, which were negatively associated with BMI-Z and WC. Similar results were reported in a cross-sectional study, where children with obesity had lower diurnal cortisol levels and an inverse association with BMI Z-score [43]. The authors suggested that low cortisol levels impair the mobilization of energy stores, as high concentrations are necessary for this process.

In contrast, a longitudinal study found that BMI at age 9 positively predicts cortisol excretion at age 12, suggesting that greater cortisol production is a consequence rather than a cause of increases in childhood BMI [44]. Girls with obesity had significantly higher salivary cortisol concentrations in all 5 measurements taken throughout the day than the normal weight group [45]. However, a systematic review with children showed a nonsignificant association of morning salivary cortisol concentration with BMI/BMI Z-score [46]. In a cohort of children and adolescents with obesity (BMI-Z 2.62 ± 0.51), serum cortisol was weakly associated with components of the metabolic syndrome, and the authors suggest that these findings do not support a major role for cortisol in the development of metabolic syndrome [43].

While elevated cortisol concentrations are associated with long-term deleterious effects, hypocortisolism in chronically stressed individuals may promote greater vulnerability to stress-related bodily disorders [47]. Both conditions are associated with long-term diseases [48]. These inconsistencies could be attributed to variability in stress levels in individuals and different cortisol measurement methods among studies [46].

In addition, the method of cortisol analysis may contribute to the discrepancies found. Salivary and hair cortisol are free, while most cortisol in the blood is carried by transport proteins. Salivary cortisol is linked to circadian rhythm stress-induced conditions, whereas hair cortisol reflects long-term cortisol concentrations [46]. The consequence of different methods is the difficulty of comparing findings across different studies, necessitating cautious interpretation of results.

The hypothalamic-pituitary-adrenal interacts with other systems to regulate a variety of behavioral, endocrine, metabolic, immune, and cardiovascular functions [49]. In addition to promoting changes in glucose metabolism and insulin resistance [50], it affects hypothalamic neuropeptides [51]. The ability to elevate DHEAS in early life may represent an adaptive neurobiological mechanism for regulating adrenocortical function and mitigating some of the negative effects of chronic stress [52-54]. In this sense, although our study does not demonstrate higher DHEA and cortisol concentrations in children with obesity, it presents symmetrical effects, with lower concentrations of both hormones. Few studies measure both hormones together, and while cortisol is regulated by adrenocorticotropic hormone, it has been described that adrenocorticotropic hormone also stimulates DHEA production [16], which may contribute to the lower concentrations observed. However, the complex nature of obesity appears to have varied effects on hormones, and other factors, such as cortisol collection time, the source of cortisol (hair, blood, urine, or saliva), and stress level, may explain, at least in part, the discrepancies in cortisol and DHEA concentrations observed in children with obesity.

In this study, no differences were found in energy consumption between children with and without obesity. One of the hypotheses for this result is the fact that children with obesity consume foods with greater energy density, while children with normal weight consume foods with greater nutritional density. Other potential explanations include reduced physical activity levels in children with obesity and the possibility of the control children developing obesity over time, given their similar intake of ultra-processed foods and calories. Nonetheless, a higher proportion of food addiction was observed in children with obesity, with a positive association found between food addiction and BMI-Z, body weight, and the consumption of total calories and lipids. These findings suggest higher energy and lipid consumption, as well as altered anthropometric parameters, in the presence of food addiction. These data are in line with previous studies [55-58].

Poor diet quality is associated with the prevalence of obesity [59]. Ultra-processed foods, high in calories, sugar, fat, and salt, contribute almost 60% of total calories and 90% of added sugars in US diets [60]. These foods are particularly prevalent in rural and low-income communities, where access to healthy food is limited and ultra-processed foods are often cheaper and more convenient [61]. Cognitive and emotional factors are important aspects that also play a significant role in eating behavior and the increased desire for highly palatable foods. A recent cohort study of 9- and 10-year-old children showed that proinflammatory diets (ie, high in saturated fats) increase neuroinflammation in reward-related brain regions, leading to even less healthy eating and contributing to obesity [62]. Food addiction may act as a partial mediator in the stress-obesity pathway, likely resulting from one or many of the biologically associated pathways [63].

The present study is one of the few that examined DHEA and morning salivary cortisol concentrations together and their associations with anthropometrics, biochemical, and metabolic profile, stress, and food addiction in children with obesity. However, several limitations should be acknowledged. Given the cross-sectional design, no causal relationships can be established to indicate impaired adrenal gland function in children with obesity. The level of physical activity was not assessed, which could be relevant to both obesity and stress. The pubertal staging was not performed, though the groups were matched for age and sex to minimize bias. DHEA is an androgenic steroid and has been associated with pubertal development. Thus, increased DHEA could be associated with earlier pubertal development by pubarche [64], but lower concentrations were observed in children with obesity in the present study. Additionally, since pubertal stage can influence steroid hormone levels, the interpretation of the results should be made with caution. Moreover, the small sample size and short sampling time limited additional findings. Food consumption assessments conducted with children may not accurately reflect habitual intake, as quantities might have been overestimated or underestimated.

In conclusion, lower cortisol and DHEA concentrations were found in children with obesity in comparison to the control group, as well as the negative correlation of these hormones with anthropometric parameters. Furthermore, children with obesity displayed impaired glucose and lipid metabolism, as well as a higher prevalence of food addiction, in comparison to their counterparts with normal weight. The combination of these findings in such a young population could potentially lead to adverse health outcomes in the long term, highlighting the need for public measures to address childhood obesity.

## Data Availability

Original data generated and analyzed during this study are included in this published article, but some restrictions apply to the availability of all data to preserve patient confidentiality. They may be accessed through the corresponding author upon reasonable request.
